# Incidental paraganglioma of the urinary bladder: A case series highlighting a rare entity and a urologist’s nightmare

**DOI:** 10.14440/bladder.2024.0064

**Published:** 2025-06-24

**Authors:** Yassir Mehmood, Rahul Gupta, Arti Mahajan, Yaser Rahman, Kshitij Gupta

**Affiliations:** 1 Department of Urology and Renal Transplant, Government Medical College, Jammu 180001, Jammu and Kashmir, India; 2 Department of Anesthesia and Critical Care, Government Medical College, Jammu 180001, Jammu and Kashmir, India; 3 Department of Urology, All India Institute of Medical Sciences, Jammu 184120, Jammu and Kashmir, India; 4 Intern Department of Surgery, Acharya Shri Chander College of Medical Sciences and Hospital, Jammu 180017, Jammu and Kashmir, India

**Keywords:** Pheochromocytoma, Urinary bladder tumor, Chromaffin cell tumor, Hematuria

## Abstract

**Background:**

Pheochromocytomas originate from chromaffin cells of the adrenal medulla, resulting in excessive production of catecholamines and bizarre clinical symptoms. An extra-adrenal pheochromocytoma is called a paraganglioma. Urinary bladder paraganglioma represents an uncommon tumor that accounts for <0.05% of all bladder tumors.

**Case presentation:**

Presented here are three cases of incidental urinary bladder paraganglioma, all having hematuria as the primary symptom. During transurethral resection of bladder tumor, blood pressure fluctuated intensely with profuse bleeding, leading to the abandonment of the procedure. The patients were later diagnosed as having paraganglioma, and then, they were subjected to systemic evaluation and definitive treatment.

**Conclusion:**

Although bladder paraganglioma is a rare condition, it should be strongly suspected if a patient who has no definitive symptoms of the disease developed a sudden hemodynamic change at the very start of the procedure.

## 1. Background

Pheochromocytoma represents a rare tumor derived from chromaffin cells. Extra-adrenal pheochromocytoma, or paraganglioma, accounts for 10 – 15% of pheochromocytoma cases. Among them, paragangliomas of the urinary bladder (PUB) are uncommon, constituting 6 – 10% of all extra-adrenal cases.^1^ Similar to other paragangliomas, PUB derives from chromaffin cells of the sympathetic nervous system. PUB is a rare tumor, first reported by Zimmerman *et al*.^2^ in 1953. While 1 – 25% of pheochromocytomas occur outside the adrenal gland, the incidence of PUB is <0.05%.^3^ In our clinical practice spanning a time of period of over 10 years, we treated more than 900 cases of bladder tumors, but only three patients were diagnosed with paraganglioma on histopathological evaluation, with an incidence of 0.003%.

## 2. Case presentation

The three PUB cases are individually presented in the following sections.

### 2.1. Case 1

A 40-year-old male presented with primary complaints of abdominal pain, burning micturition, and hematuria for the past month. Systemic examination showed that the patient was pale and the examination results were otherwise normal. Ultrasound of the abdomen revealed a lesion of mixed opacity, along the superior wall of the urinary bladder. The patient was clinically stable and scheduled for transurethral resection of bladder tumor (TURBT). Intraoperatively, a solid growth was identified along the anterior bladder wall, which began to bleed profusely from the outset of the resection. This was accompanied by significant fluctuations in blood pressure during the resection. Despite the hemodynamic instability, the procedure was successfully completed, and hemostasis was achieved. Postoperatively, the patient required three units of blood transfusion and was managed in the intensive care unit for 2 days.

**Figure 1:**
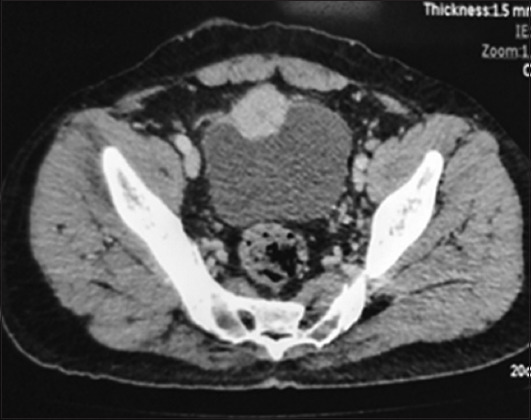
Contrast-enhanced CT showing a hyperdense mass along the anterior wall of the urinary bladder. Abbreviations: CT: Computed tomography.

**Figure 2:**
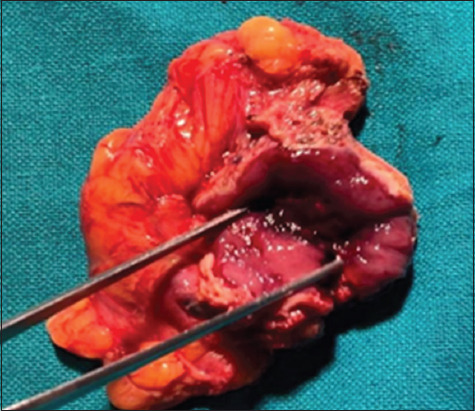
Resected specimen (internal view) showing tumor.

Subsequent histopathological examination (HPE) revealed paraganglioma. Further investigation with contrast-enhanced computed tomography (CECT) found a hyperdense lesion along the anterior bladder wall (Figure 1). Urinary metanephrines were elevated. The patient was then medically optimized with both alpha- and beta-adrenergic blockers and planned for partial cystectomy. He underwent laparoscopic partial cystectomy with wide local excision of the tumor (Figure 2). The procedure was uneventful, and the patient remained normotensive throughout the perioperative period. He is currently on regular follow-up, remains asymptomatic, and has shown no sign of recurrence.

### 2.2. Case 2

A 35-year-old male, a chronic smoker, presented with complaints of burning micturition and hematuria for 2 months. On examination, the patient’s blood pressure was at 160/95 mmHg, for which he was put on antihypertensive medication during hospitalization. The rest of the systemic examination was normal. Ultrasound imaging showed a 3 × 3 cm mass lesion located along the posterior wall of the urinary bladder. Following medical optimization, the patient underwent TURBT. Intraoperatively, as soon as the lesion was manipulated, there were marked fluctuations in blood pressure. The tumor, situated on the posterior wall, had excessive bleeding, more than is typically observed with conventional bladder tumors. HPE revealed paraganglioma. A CECT exhibited a heterogeneously enhancing lesion along the posterior wall of the urinary bladder. The patient was scheduled for partial cystectomy; however, he was subsequently lost to follow-up.

### 2.3. Case 3

**Figure 3:**
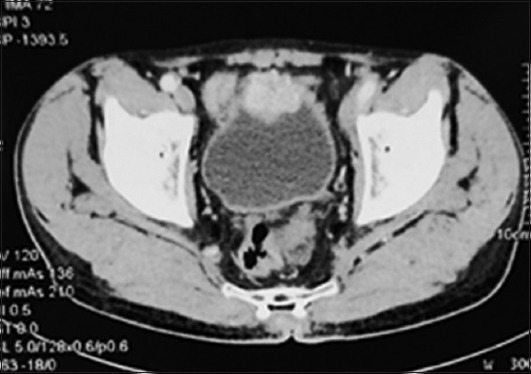
Contrast-enhanced CT exhibiting a hyperdense mass at the anterior wall of the urinary bladder. Abbreviations: CT: Computed tomography

A 63-year-old male, a chronic smoker, presented to a private clinic with the chief complaint of hematuria. Initial evaluation revealed a bladder mass at the superior wall of the urinary bladder, for which he underwent TURBT. However, upon the first contact with the lesion, the patient’s blood pressure surged tremendously, prompting the surgical team to abort the procedure after obtaining a biopsy. The HPE report revealed the diagnosis of paraganglioma. The patient was evaluated further in our center. A CECT scan showed a hyperdense mass lesion sized 3.5 × 3.5 cm at the superior bladder wall with heterogenous enhancement (Figure 3). Urinary metanephrine levels were significantly elevated.

**Figure 4:**
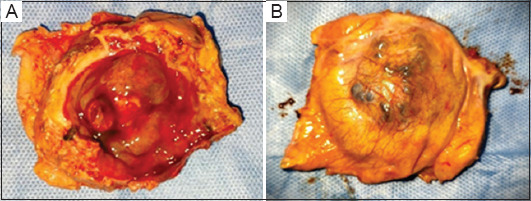
Resected partial cystectomy specimen showing bladder tumor. (A) Internal view. (B) External view.

The patient’s initial biopsy suggested paraganglioma. In consultation with an endocrinologist, the patient was given alpha blockers followed by beta blockers, before definitive surgery. He then received laparoscopic partial cystectomy with extensive excision of the lesion (Figure 4).

**Figure 5:**
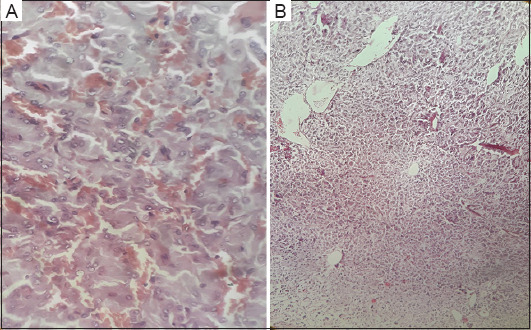
Histological features suggestive of paraganglioma. (A) Magnified image of the pheochromocytoma specimen showing chromaffin cells arranged in clusters. (B) Low-power histopathological images of the pheochromocytoma specimen showing nesting pattern. (A) Magnification: 40x, Scale bar: 40 μm; (B) Magnification: 10x, scale bar: 200 μm.

The postoperative stay was uneventful, and the patient was discharged on postoperative day 3. HPE report showed a capsulated lesion with cells arranged in a *zellballen* pattern. These cells were monomorphic, with abundant eosinophilic to amphophilic cytoplasm, and a nucleus containing powdery chromatin. These features were suggestive of paraganglioma (Figure 5).

## 3. Discussion

Bladder paraganglioma is a rare, non-epithelial neuroendocrine neoplasm arising from autonomic nervous tissue, specifically chromaffin cells, and accounts for <0.05% of all bladder tumors.^2^ The peak incidence occurs between the third and fifth decades of life, with a male-to-female ratio of 1.07:1. Clinically, PUB usually presents with palpitations, sweating, headache, and hypertension (paroxysmal/sustained) during micturition or overdistention of the bladder.^3^ These tumors are often metabolically active, demonstrating elevated catecholamine levels. Patients with such symptoms should be highly suspected of having the condition. Imaging modalities that aid in diagnosis include ultrasonography, computed tomography (CT), magnetic resonance imaging (MRI), and metaiodobenzylguanidine scanning.^4^ More advanced nuclear imaging modalities, such as positron emission tomography (PET)-CT Gallium 68 DOTATATE scan, have proven highly effective in identifying metastatic disease, while 18F-fludeoxyglucose and 18F-dopamine PET scans offer greater sensitivity and specificity for the diagnosis of abdominopelvic paragangliomas.^5^

In cases of bladder lesions, the location of the lesion, size, and shape can be determined through cystoscopic examination.^6^ Unfortunately, PUB tends to masquerade as bladder cancer and is often misdiagnosed, especially those non-functional tumors with no symptoms.^7^ One study reported that 61.6% of patients with PUB were misdiagnosed prior to pathological diagnosis, and <30% were accurately diagnosed preoperatively.^8^ These findings were consistent with our experience. In all three of our cases, clinical suspicion only arose intraoperatively, when patients experienced severe blood pressure fluctuations during tumor manipulation, despite preoperative absence of classical symptoms suggestive of pheochromocytoma.

As reported in our series, an intraoperative hypertensive crisis should immediately raise suspicion for paraganglioma, especially in the absence of prior symptoms. Similar findings were reported by Menon *et al*.^9^ in 2014, who reported that only 7% of patients had elevated preoperative catecholamine levels. With a high index of clinical suspicion, these atypical tumors can occasionally be diagnosed preoperatively.

In the preoperative cases where functional PUB is suspected and catecholamine levels are elevated (in 7 – 30% of cases),^8^,^9^ endocrinological consultation is essential to avoiding detrimental effects of sudden spikes of catecholamine secretion intraoperatively.^10^ However, in our cases, none of the patients had classical clinical features of pheochromocytoma, and the diagnosis was only considered intraoperatively due to extreme fluctuations of blood pressure, and later histopathologically confirmed.

Following definitive diagnosis, the treatment of choice for organ-confined disease is either a partial cystectomy or radical cystectomy and chemoradiation in cases of advanced disease.^5^,^11^,^12^ In our series, all three patients were diagnosed after transurethral resection of the tumor and had organ-confined disease. They subsequently underwent laparoscopic partial cystectomy with wide margins, and final HPE confirmed paraganglioma with a margin-free status in all cases.

Prognosis depends on factors such as the size of the lesion, the presence of metastasis, and associated familial endocrinopathies. Up to 30% of paraganglioma of urinary bladder may be familial, particularly in younger patients, and may be associated with syndromes such as multiple endocrine neoplasia type 2 and von Hippel–Lindau disease. In literature, six genes have been found to be associated with familial predisposition, *i.e*., *RET*, *VHL*, *NF1*, *SDHB*, *SDHC*, and *SDHD*.^13^ Genetic testing is recommended for patients younger than 50 years, those with paraganglioma, or those with multiple tumors.

Patients with paraganglioma usually require prolonged follow-up, as local recurrence can occur in approximately 15% of cases, even after margin-negative resection. MRI or CECT is recommended as an ideal imaging follow-up every 1 – 2 years.^5^,^11^,^14^ All three of our patients are on regular follow-up at 6-month intervals and remain disease-free on radiological study, with a maximum follow-up period of 3 years.

## 4. Conclusion

Although PUB is a rare tumor, it should be considered in patients with hematuria, headache, micturition syncope, and palpitations. However, it can mimic more common bladder tumors, especially when typical catecholamine-related symptoms are absent. Intraoperative findings such as profuse bleeding and sudden, severe hemodynamic instability during endoscopic resection should prompt immediate suspicion of PUB. In such cases, it is prudent to abort the procedure after obtaining a biopsy and achieving hemostasis, followed by systematic evaluation and definitive treatment.

## Data Availability

Data will be made available on reasonable request from the corresponding author.
